# Development of 20 Microsatellite Markers for *Solenocera crassicornis* and Their Cross-Species Application in *Solenocera melantho*

**DOI:** 10.3390/ijms13079218

**Published:** 2012-07-23

**Authors:** Shufang Liu, Hongbo Liu, Lin Lin, Yanjiao Yuan, Fangqun Dai, Zhimeng Zhuang

**Affiliations:** 1 Key Laboratory for Fishery Resources and Eco-Environment, Yellow Sea Fisheries Research Institute, Chinese Academy of Fishery Sciences, Qingdao 266071, China; E-Mails: liusf@ysfri.ac.cn (S.L.); liuhongbo23@163.com (H.L.); jiaoyy1987@163.com (Y.Y.); dai@ysfri.ac.cn (F.D.); 2 Key Laboratory of Mariculture, Ministry of Agriculture, Dalian Ocean University, Dalian 116023, China; 3 South China Sea Fisheries Research Institute, Chinese Academy of Fishery Sciences, Guangzhou 510300, China; E-Mail: linlinbio@163.com; 4 College of Fisheries and Life Science, Shanghai Ocean University, Shanghai 201306, China

**Keywords:** *Solenocera crassicornis*, *Solenocera melantho*, microsatellite marker, cross-species amplification

## Abstract

Twenty microsatellite markers were isolated and characterized for *Solenocera crassicornis* from a (GT)13-enriched genomic library. Their polymorphisms were investigated using 44 wild individuals from the South Yellow Sea. Our investigation revealed that all the markers were polymorphic. The number of alleles per locus varied from 6 to 19 with an average of 12.35. The observed and expected heterozygosities ranged from 0.400 to 0.977 and from 0.609 to 0.940, with averages of 0.788 and 0.859, respectively. Four loci significantly deviated from Hardy-Weinberg equilibrium after Bonferroni’s correction. Cross-species amplification was also conducted in *Solenocera melantho* collected from the East China Sea. The result showed that 14 loci could be amplified from *Solenocera melantho* DNAs. These polymorphic markers would be useful for assessment of genetic variation and population structure of *S. crassicornis* and *S. melantho*.

## 1. Introduction

*Solenocera crassicornis* is an important prawn species in fishery resource and is widely distributed in the adjacent waters of Singapore, Indonesia, India, and the South Yellow Sea and the East Sea of China [1]. *S. crassicornis*, along with *Parapenaeopsis hardwickii*, *Trachypenaeus curvirostris*, *Metapenaeopsis dalei*, and *Palaemon gravieri*, constitute the five major fishing prawn species of the Yellow Sea and East China Sea of China. Their catches occupied 80%~90% of the total production in the waters’ dragged prawn resources, and created high economic value [2–4]. In addition, *S. crassicornis* occupies an important niche within ecosystems, constituting a large proportion of food for fish [3]. Unfortunately, in recent years, the resource of *S. crassicornis* has been gradually declining possibly due to over-exploitation and ocean environmental deterioration [5]. As a result, it is necessary and important to carry out research on *S. crassicornis* to promote the effective conservation and sustainable development of this prawn species. At present, there are few reports on study of *S. crassicornis* at home and abroad, except for on the fishery and biology [3,4,6].

Species identification and population genetics assessment may lead to a better understanding of the effects of over-exploitation and environmental change on the fishery stocks. The microsatellite markers are highly polymorphic and the data is relatively easy to statistics, which is proven to be an extremely valuable tool for genetic studies and conservation and management of genetic resources [7–10].

Here, we developed the microsatellite loci in *S. crassicornis* and characterized the microsatellite markers by genotyping 44 individuals sampled from a wild population. Additionally, cross-species amplification was carried out in 10 individuals of *S. melantho* to determine the potential for cross utility. This study would provide a technical support to investigate and evaluate the status of the genetic resources of *S. crassicornis*.

## 2. Results and Discussion

Twenty microsatellite markers were isolated and characterized for *S. crassicornis*. The numbers of alleles per locus ranged from 6 to 19 with an average of 12.35. It revealed that all the markers were highly polymorphic. The observed and expected heterozygosities ranged from 0.400 to 0.977 and from 0.609 to 0.940, with averages of 0.788 and 0.859, respectively (Table 1). Four loci deviated from Hardy-Weinberg equilibrium in the tested population after Bonferroni’s correction (adjusted *P*-value < 0.002), which might be caused by existence of null alleles. Upon the analysis, the following four loci: ZG-18, ZG-38, ZG-47 and ZG-90, were predicted to have null alleles, and they deviated from the HWE. Based on the analysis of the Bonferroni’s correction, there are three loci pairs (ZG-1/ZG-6, ZG-6/ZG-61 and ZG-10/ZG-85) are in linkage disequilibrium (*P*-value < 0.002).

Cross-species amplification test showed that 14 of these 20 loci were successfully cross-amplified in the related species of *S. melantho* (Table 1). It confirmed that microsatellite markers developed in *S. crassicornis* could be used effectively for related prawn species.

## 3. Experimental Section

### 3.1. DNA Extraction

Forty-four individuals of *S. crassicornis* and ten individuals of *S. melantho* were collected from the South Yellow Sea and the East China Sea, respectively. Samples were preserved in alcohol and stored at −20 °C until DNA extraction. Genomic DNA was extracted from muscle tissue using a standard traditional the phenol-chloroform procedure.

### 3.2. Microsatellite-Enriched Library Construction

Microsatellite-enriched library was constructed using the FIASCO (Fast Isolation by AFLP of Sequences Containing Repeats) method [11] with minor betterment. Genomic DNA was digested with *Mse* I (New England Biolabs, USA) at 37 °C for 3 h, and the digested DNA (10 μL) was ligated to *Mse* I adaptor (5′-TAC TCA GGA ACT CAT-3′/5′-GAC GAT GAG TCC TGA G-3′). Linker-ligated DNA was amplified in a 25 μL reaction mix using the adapter-specific primer (5′-GAT GAG TCC TGA GTA A-3′). Polymerase chain reaction (PCR) conditions were as follows: 20 cycles at 94 °C for 30 s, 53 °C for 1 min, 72 °C for 1 min. The PCR products were purified using DNAmate (TaKaRa, Japan) and hybridized to a biotin labeled (GT)13 probe. The mixture was denatured at 94 °C for 5 min, then at 53 °C for 15 min. The hybrids were captured with streptavidin-coated magnetic beads (Promega, USA). Unhybridized DNA was washed away, and the remaining DNA was eluted from the magnetic beads and amplified using the adaptor-specific primer and the above PCR program. Following purification, DNA fragments ranging from 500 bp to 1000 bp were selected by separation on 1.5% agarose gels. The fragments were ligated to pMD18-T vectors (TaKaRa, Japan), and transformed into *Escherichia coli* DH5α competent cells to produce a microsatellite-enriched library.

### 3.3. Isolation of Microsatellite-Containing DNA Fragments

After amplifying with (GT)13 and M13 primers, 200 positive clones were obtained. The positive clones were sequenced on an ABI 3730 automated DNA sequencer (Applied Biosystems, USA). The sequencing data were scanned using the software SSRHunter V1.3 [12]. Ninety pairs of primers were designed using PRIMER PREMIER5 (Premier Biosoft International, USA) and tested for polymorphism with six *S. crassicornis* individuals.

### 3.4. PCR Amplification and Genotyping

After preliminary screening, only 20 polymorphic microsatellite loci were tested on a sample of 44 individuals. PCR for all loci was performed separately in a 25 μL reaction volume containing 0.4 μM of each primer, 0.2 mM dNTPs, 2 mM MgCl_2_, 1× PCR buffer, 1 U Taq polymerase (Fermentas, Canada) and 50–100 ng DNA. Amplification was carried out with the following thermal profile: 94 °C for 5 min, followed by 35 cycles of 94 °C for 45 s, optimal annealing temperature (Table 1) for 45 s, and 72 °C for 45 s, and a final extension step at 72 °C for 10 min. PCR products were separated on 6% denaturing polyacrylamide gels and visualized by silver-staining.

### 3.5. Genetic Data Analysis

Allele sizes were estimated according to the pBR322/*Msp* I marker (Tiangen, China). The variability at each locus was measured in terms of number of alleles, expected heterozygosity and observed heterozygosity, and the Hardy-Weinberg equilibrium and linkage disequilibrium test were conducted using GENEPOP 4.0 [13]. Null allele frequencies were calculated using Micro-Checker 2.2.3 [14]. Significance criteria of all multiple tests were corrected following sequential Bonferroni’s correcting [15].

## 4. Conclusions

In the present study, microsatellite-enriched genomic library of *Solenocera crassicornis* was constructed and a total of 20 polymorphic microsatellite DNA markers shown as the first set of microsatellite loci were isolated and characterized. Most of these markers could be amplified successfully in *Solenocera esculenta*. These loci will prove helpful in the management of fisheries and in the design of conservation strategies.

## Figures and Tables

**Table 1:** Characterization of microsatellite loci developed for *Solenocera crassicornis* and *Solenocera melantho*.

Loci	Genbank accession No.	Repeat motif	Primer sequence (5′–3′)	Size range (bp)	*T*m (°C)	*N*A	*H*O	*H*E	*P*HW	Cross-amplified in *Solenocera melantho*
ZG-1	JQ954744	(AC)5…(CA)8…(CA)6	F: TTGCCTTCCAAGCACATTCA R: ATCCCTGTTAGCTCATTCCACA	340–430	55	16	0.9318	0.9135	0.0927	+
ZG-6	JQ954745	(AC)6…(AC)7…(AC)7…(AC)8	F: AGGTTTATTTGGTTTATG R: ATATCTCGCTATCTCATT	240–480	55	14	0.8974	0.8944	0.1973	+
ZG-7	JQ954746	(TC)7…(CA)14…(AC)36…(AG)11	F: TCTGTATTTCGTCTTGGA R: GATAGCGGGTTGAGGT	300–410	55	19	0.9545	0.9122	0.8769	+
ZG-9	JQ954747	(TG)5…(GT)5	F: TACACGAATGAGGCATAG R: GTGGTAACAGACAGACAATC	300–370	55	15	0.8181	0.8657	0.1215	+
ZG-10	JQ954748	(CA)6…(AT)6	F: TCGGAAGTAACATTCAGGAC R: GGAAGGAAATTCTACGCTAT	330–360	55	7	0.8409	0.7996	0.8719	+
ZG-18	JQ954749	(AT)5…(AC)5…(CA)5…(AC)5…(CA)29	F: CTTTATCTGGTCGGGTTT R: GACGAAGTGAATAGACTGTG	310–500	55	19	0.4000	0.9395	0.0000*	−
ZG-20	JQ954750	(TG)7…(GT)5	F: AAAATGGAATGCGATAGAT R: TTATAGCGAACCAACACCT	230–260	45	11	0.7954	0.8853	0.0149	+
ZG-27	JQ954751	(AC)7	F: GCTTCTCAAGGGAGGCACA R: GCGGGAAGGATGGAGGTA	270–310	55	6	0.7500	0.7526	0.3399	−
ZG-29	JQ954752	(AC)17	F: GATATGGCGGTTGAGTGA R: TACGTGGTTTATGTTGCTTA	310–350	55	10	0.8181	0.8791	0.3072	+
ZG-36	JQ954753	(CA)38	F: AGAGTGACGGTCAAACTGA R: TAAACGCATTAGGAGACG	300–400	55	17	0.8809	0.9185	0.2256	−
ZG-38	JQ954754	(GT)12	F: GTCTGCACGGGATTTGTTCT R: CGCTCGTCCAATTAGGGTAT	260–290	55	7	0.4545	0.7988	0.0000 *	−
ZG-40	JQ954755	(AGAC)18	F: ATTGCGTTGGAAATGTATC R: GTCCCTTTTATTGTCTATCTGT	300–450	55	15	0.8409	0.8874	0.1864	−
ZG-47	JQ954756	(AC)5…(AC)6…(CA)8…(AC)5	F: TTTTGTATTCTTGTTCTGGAT R: TGATTCGTTGTTTCATTTG	180–210	55	10	0.5814	0.8768	0.0000*	+
ZG-61	JQ954737	(AC)5…(CT)5	F: GACGAGGAACAAATCAGA R: TTGAAGAATAAGAGGGACT	320–410	45	12	0.9772	0.8892	0.9555	+
ZG-66	JQ954738	(AC)14	F: TTCCATCCTATTTCTACTG R: ATAAGACGTTTACCTACAT	280–330	55	15	0.9772	0.9148	0.9964	+
ZG-67	JQ954739	(AT)5…(TG)5	F: TGGAAGTAACAACTAAACTTTG R: CAACCCAGAGGTGTCAGA	330–360	55	7	0.5909	0.6091	0.2619	+
ZG-71	JQ954740	(CA)61	F: AAAGGCTGAAATCAAGAAG R: GAGGAAGAATGAGCGTTAG	220–300	45	13	0.9772	0.8902	0.9736	+
ZG-75	JQ954741	(AC)33	F: CCGAACTGGCACCACTAT R: AACGGATTCCTATTACAGACAA	210–260	55	12	0.8863	0.8605	0.0513	−
ZG-85	JQ954742	(TG)51…(GT)6	F: AATGACAACTCTACAGGCTA R: TCAATTCCAAGTGAATGC	310–400	55	16	0.909	0.9192	0.4320	+
ZG-90	JQ954743	(GT)13	F: ACACTTTCTACATTCCAC R: GTCACTCATCCATTCAC	180–200	45	6	0.4772	0.7816	0.0003*	+

*T*_m_: annealing temperature (°C); *N*_A_: number of alleles; *H*_O_: observed heterozygosity; *H*_E_: expected heterozygosity; *P*_HW_: *P* value for exact test for Hardy-Weinberg equilibrium (HWE);

* indicated departure from HWE after Bonferroni’s correction (*P* < 0.002); +: successful in cross-species amplification; −: unsuccessful in cross-species amplification.

## References

[1] Liu R.Y. (1959). Regional character of economic shrimp species in Yellow Sea and East China Sea (in Chinese). Oceanol. Limnol. Sin.

[2] Song H.T., Yao G.Z., Yu C.G., Xue L.J. (2003). Species composition and quantitative distribution of shrimps in East China Sea (in Chinese). Acta Oceanol. Sin.

[3] Song H.T., Yao G.Z., Yu C.G., Xue L.J. (2003). Study on the biomass distribution and biological characteristics of *Solenocera crassiocornis* in East China Sea (in Chinese). J. Zhejiang Ocean Univ. Sci. A.

[4] Ye S.Z., Zhang Z.L., Ye Q.T. (2012). Quantitative distribution and biological characteristics of *Solenocera crassicornis* in northeast Fujian outer-sea (in Chinese). South China Fish. Sci.

[5] Cheng J.H., Dai G.J., Ling J.Z., Liu M. (2003). Discussion on management countermeasure of economic shrimps in East China Sea (in Chinese). China Fish.

[6] Sukumaran K.K. (1978). Studies on the fishery and biology of *Solenocera crassicornis* (H. Milne Edwards) from Bombay waters. J. Mar. Biol. Assoc. India.

[7] Yuan Y.J., Liu S.F., Bai C.C., Liu H.B., Zhuang Z.M. (2012). Isolation and characterization of new 24 microsatellite DNA markers for golden cuttlefish (*Sepia esculenta*). Int. J. Mol. Sci.

[8] Liao M.J., Wang Y.G., Rong X.J., Zhang Z., Li B. (2011). Development of new microsatellite DNA markers from *Apostichopus japonicus* and their cross-species application in *Parastichopus parvimensis* and *Pathallus mollis*. Int. J. Mol. Sci.

[9] Adams B.K., Hutchings J.A. (2003). Microgeographic population structure of brook charr: A comparison of microsatellite and mark-recapture data. J. Fish Biol.

[10] Wennevik V., Skaala O., Titov S., Studyonov I., Naevdal G. (2004). Microsatellite variation in populations of Atlantic salmon from north Europe. Environ. Biol. Fishes.

[11] Zane L., Bargelloni L., Patarnello T. (2002). Strategies for microsatellite isolation: A review. Mol. Ecol.

[12] Li Q., Wan J.M. (2005). SSRHunter: Development of a local searching software for SSR sites (in Chinese). Hereditas.

[13] Rousset F. (2008). Genepop’007: A complete re-implementation of the Genepop software for Windows and Linux. Mol. Ecol. Res.

[14] Van Oosterhout C., Hutchinson W.F., Wills D.P.M., Shipley P. (2004). MICRO-CHECKER: Software for identifying and correcting genotyping errors in microsatellite data. Mol. Ecol. Notes.

[15] Rice W.R. (1989). Analyzing tables of statistical tests. Evolution.

